# The Double-Edged Sword of T1-Mapping in Systemic Sclerosis—A Comparison with Infectious Myocarditis Using Cardiovascular Magnetic Resonance

**DOI:** 10.3390/diagnostics10050335

**Published:** 2020-05-24

**Authors:** George Markousis-Mavrogenis, Loukia Koutsogeorgopoulou, Gikas Katsifis, Theodoros Dimitroulas, Genovefa Kolovou, George D. Kitas, Petros P. Sfikakis, Sophie I. Mavrogeni

**Affiliations:** 1 Cardiology Department, Onassis Cardiac Surgery Center, 17674 Athens, Greece; georgemm32@gmail.com (G.M.-M.); genovefa@kolovou.com (G.K.); 2 Pathophysiology Dpt., Laikon Hospital, 11527 Athens, Greece; lukia.km@gmail.com; 3 Rheumatology Unit, Navy Hospital, 11521 Athens, Greece; katsifisg@yahoo.gr; 4 Internal Medicine Dpt., Aristotle University of Thessaloniki, 54124 Thessaloniki, Greece; dimitroul@gmail.com; 5 Arthritis Research UK Epidemiology Unit, University of Manchester, Manchester M13 9PL, UK; gkitas@hygeia.gr; 6 First Department of Propaedeutic and Internal Medicine, Laikon Hospital, Athens University Medical School, 11527 Athens, Greece; psfikakis@med.uoa.gr; 7 National and Kapodistrian University of Athens, 15772 Athens, Greece

**Keywords:** scleroderma, cardiovascular magnetic resonance, myocardial fibrosis, myocardial edema, parametric, T2-mapping, ECV, Lake Louise criteria

## Abstract

Aims: T1-mapping is considered a surrogate marker of acute myocardial inflammation. However, in diffuse cutaneous systemic sclerosis (dcSSc) this might be confounded by coexisting myocardial fibrosis. We hypothesized that T1-based indices should not by themselves be considered as indicators of myocardial inflammation in dcSSc patients. Methods/Results: A cohort of 59 dcSSc and 34 infectious myocarditis patients was prospectively evaluated using a 1.5-Tesla system for an indication of suspected myocardial inflammation and was compared with 31 healthy controls. Collectively, 33 (97%) and 57 (98%) of myocarditis and dcSSc patients respectively had ≥1 pathologic T2-based index. However, 33 (97%) and 45 (76%) of myocarditis and dcSSc patients respectively had ≥1 pathologic T2-based index. T2-signal ratio was significantly higher in myocarditis patients compared with dcSSc patients (2.5 (0.6) vs. 2.1 (0.4), *p* < 0.001). Early gadolinium enhancement, late gadolinium enhancement and T2-mapping did not differ significantly between groups. However, both native T1-mapping and extracellular volume fraction were significantly lower in myocarditis compared with dcSSc patients (1051.0 (1027.0, 1099.0) vs. 1120.0 (1065.0, 1170.0), *p* < 0.001 and 28.0 (26.0, 30.0) vs. 31.5 (30.0, 33.0), *p* < 0.001, respectively). The original Lake Louise criteria (LLc) were positive in 34 (100%) myocarditis and 40 (69%) dcSSc patients, while the updated LLc were positive in 32 (94%) and 44 (76%) patients, respectively. Both criteria had good agreement with greater but nonsignificant discordance in dcSSc patients. Conclusions: ~25% of dcSSc patients with suspected myocardial inflammation had no CMR evidence of acute inflammatory processes. T1-based indices should not be used by themselves as surrogates of acute myocardial inflammation in dcSSc patients.

## 1. Introduction

Systemic sclerosis (SSc) is an autoimmune disease characterized by microvascular abnormalities, inflammation and fibrosis of all organs including the heart [1]. Despite therapeutic advances, SSc patients are still 2.7–3.5 times more likely to die compared with the general population [2], with cardiac involvement being an important contributor [3]. Primary cardiac disease in SSc patients is either caused by coronary microvasculopathy or by primary myocardial inflammation, with the end effect being myocardial fibrosis [4]. The early detection of myocardial inflammation in particular is of great clinical significance in SSc, because, in contrast to microvasculopathy, it is an acute process and should be managed with short-term immunosuppressive treatment [4]. Furthermore, the prognosis of SSc patients is poor with an event rate of 28% within 22.5 months of follow up and is associated with the degree of cardiac inflammation and fibrosis [5]. 

Cardiovascular magnetic resonance (CMR) has already been successfully used for evaluating cardiac involvement in SSc [6,7,8]. CMR can detect myocardial oedema and replacement fibrosis using T2-weighted short tau imaging (STIR-T2) and late gadolinium enhancement (LGE) imaging respectively [9,10]. Native T1- and T2-mapping are highly sensitive to myocardial water content and more accurate than STIR-T2 in the detection of myocardial oedema [11,12]. T2-mapping can also make up for the propensity of STIR-T2 for identifying artefacts as nonexistent oedema [13]. Native T1-mapping and extracellular volume fraction (ECV) measurements can also act as a surrogate of diffuse fibrosis [14]. These indices are highly relevant for SSc, as cardiac fibrosis in these patients might assume both replacement and diffuse types [15]. 

However, despite the ability of CMR to adequately assess these patients, the differentiation of treatable myocardial inflammation from the end-effect of myocardial fibrosis using CMR remains elusive. We hypothesized that T1-based indices should not by themselves be considered as indicators of myocardial oedema in SSc, because they might represent expansion of the extracellular space associated with the deposition of fibrous tissue as a result of the progression of coronary microvasculopathy and not necessarily due to acute inflammation. To test this hypothesis, we aimed to evaluate a cohort of SSc patients with suspicion of myocardial inflammation using CMR in order to examine the relationship between T1- and T2-based indices and to characterize the constellation of CMR findings in this group. We also aimed to evaluate patients referred for CMR due to suspected infectious myocarditis and healthy controls for comparison. 

## 2. Materials and Methods 

### 2.1. Patients

We prospectively recruited 59 patients diagnosed with diffuse-cutaneous SSc (dcSSc) according to the 1980 American College of Rheumatology criteria [9]: 34 patients with a clinical suspicion of infectious myocarditis and 31 healthy controls. All patients were referred for CMR due to atypical chest pain, palpitations and/or shortness of breath. All controls participated voluntarily and had no objectifiable cardiovascular symptoms. The study was approved by the Onassis cardiac surgery center medical ethics committee (project identification code: 434, date: 13 August 2010). All participants provided written informed consent before their inclusion to the study. 

### 2.2. Methods

CMR was performed with a 1.5-T scanner (Ingenia, Philips Medical Systems, Best, The Netherlands). The CMR protocol included standard steady-state free-precession cine CMR, black-blood STIR-T2 images, T1-weighted spin-echo EGE CMR, and phase-sensitive inversion recovery LGE CMR as described previously [16]. A dose of 0.1 mmol/kg gadobenate dimeglumine contrast medium was injected for EGE and another 0.1 mmol/kg for LGE, according to the protocol recommended by the original journal of the American college of cardiology white paper authors [17]. 

T1-mapping was performed using a modified Look-Locker inversion recovery (MOLLI) sequence with a 3(3)5 scheme on three representative short-axis positions before and 15 min after contrast-media administration. T2-mapping was performed on three corresponding left ventricular (LV) short axes using a black-blood-prepared, navigator-gated, free-breathing hybrid gradient (echo planar imaging) and spin-echo multiecho sequence [16]. 

### 2.3. CMR Data Analysis

Global myocardial inflammation was assessed in STIR-T2 images by calculating the T2 signal intensity ratio as signal intensity of myocardium divided by signal intensity skeletal muscle [17]. Global relative enhancement was calculated by measuring myocardial signal intensity on pre- and post-contrast T1-weighted spin-echo images relative to skeletal muscle [17]. The presence and pattern of nonischemic LGE lesions were qualitatively assessed by consensus agreement of two experienced observers (SM and DM). Intra- and interobserver agreement was 0.88 and 0.85, respectively. 

Color-coded T1 and T2 maps were generated based on inline-generated, motion-corrected raw images using Philips software in three matching short axis slices. Motion-corrected T1 maps were examined for quality in raw T1 images, T1 maps and T2 maps. Endocardial and epicardial contours were manually drawn by two experienced observers (SM and DM). Global T1, ECV, and T2 values were calculated. Before the CMR examination, the hematocrit was determined in all subjects, allowing the calculation of ECV in conjunction with native and postcontrast T1-mapping measurements, using a previously described equation [18]. T2 results were obtained by fitting a two-parameter, intensity-weighted exponential model [18].

### 2.4. Statistical Analysis

Statistical analyses were performed with the software Stata SE v.15SE and R v.3.6.1. Normality testing was performed for all continuous study parameters by means of Q-Q plots and, alternatively, a histogram. Normally distributed continuous variables are presented as mean (standard deviation), not-normally distributed variables are presented as median (interquartile range) and binary/categorical variables are presented as N (%). Comparisons between three groups were carried out using one-way analyses of variance, Kruskal–Wallis tests and chi-square tests where appropriate. Post hoc testing between myocarditis and SSc patients was similarly carried out using independent sample t-tests, Mann–Whitney tests and chi-square tests where appropriate. Statistical significance was considered for *p* ≤ 0.05. The significance threshold for p-values of post hoc tests was adjusted using Benjamini–Hochberg (false discovery rate) corrections. 

### 2.5. Myocardial Inflammation According to the Original and Updated Lake Louise Criteria

Traditionally, myocarditis/myocardial inflammation is diagnosed using CMR based on the Lake Louise (JACC white paper) criteria, including STIRT2 for myocardial oedema, early gadolinium enhancement (EGE) for hyperemia/capillary leak and late gadolinium enhancement (LGE) for replacement fibrosis [17]. These criteria were recently updated to include the novel mapping-based indices, including T2-mapping for diffuse myocardial oedema, as well as native T1 mapping and extracellular volume fraction (ECV) for extracellular space expansion (due either to oedema or to fibrosis) [19]. Accordingly, binary variables where generated based on the pathologic values for the original Lake Louise criteria (oLLc) defined in the original JACC white paper (T2 ratio > 1.9, EGE > 4 and LGE > 0%), as well as the updated Lake Louise criteria (uLLc) (T1 indices: a. elevated native T1-mapping/ECV b. nonischemic LGE pattern; T2 indices: a. elevated T2-mapping b. elevated STIR-T2 myocardium to skeletal muscle ratio; diagnosis is positive if at least one index is pathologic in each of the T1 and T2 categories) [19]. We used cut-off points for normal values previously determined at our imaging center for the definition of pathologic values of novel parametric indices (native T1-mapping > 1050 ms, T2-mapping > 55 ms and ECV > 29). The calculation method for both criteria is presented in Table 1. 

### 2.6. Investigation of Between-Method Agreement

Between-method agreement for the oLLc and uLLc was assessed with Cohen’s kappa test and marginal homogeneity was assessed with McNemar’s test separately for each of the three patient groups. Raw, positive and negative agreement was calculated for each comparison and 95% confidence intervals (95% CI) for proportions and for kappa values were calculated using the bootstrap method. 

### 2.7. Feature Selection

The ncvreg package for R [20] was used to carry out minmax concave penalty (MCP) logistic regression analyses with k-fold cross-validation for discriminating between controls and myocarditis/SSc patients, in order to inform variable selection for multivariable models. All CMR indices including the oLLc/uLLc were investigated as potential features. The optimal value for the penalization term λ was determined as the value that minimizes the cross-validation error rate derived from k-fold cross-validation. The reliability of selected features was evaluated using the built-in marginal false discovery rate (mFDR), which performs better than other inference methods for penalized regression analyses [21,22]. Model predictive capacities are reported as cross-validated R^2^ values. Penalized regression analysis can overcome the disadvantages of stepwise or best subset approaches for feature selection [23] and allows for the selection of important predictors by optimizing the variance-bias tradeoff [24]. This ensures optimal external validity for the identified predictors at the cost of more biased estimates. The employed type of penalization (MCP) has been shown to be less biased towards features with larger coefficients than other penalization methods like least absolute shrinkage and selection operator (LASSO) [20,23] and was thus preferred. 

## 3. Results 

### 3.1. Baseline Characteristics

The study population consisted of 59 dcSSc patients aged 53 (13) years, 55 (93%) of which were female, with a 4 (2–10) year median duration since SSc diagnosis at the time of inclusion. The group of 34 patents with suspected infectious myocarditis had a mean age of 38 (17) years with 15 (44.1%) being female. The group of 31 healthy controls had a mean age of 39 (9) years with 12 (38.7%) being female. Regarding comorbidities, three (8%) patients with myocarditis and five (8%) patients with SSc had hypertension, while one (3%) and two (3%) had dyslipidemia respectively. No controls had known hypertension or dyslipidemia and none of the participants were smokers. Baseline characteristics with descriptive statistics and univariable statistical testing between groups are presented in Table 2. 

### 3.2. Comparison of CMR Indices between Groups 

SSc patients had a median period of 4.0 (2.0–10.0) years since diagnosis. All variables differed significantly between the three groups. In post hoc analyses comparing myocarditis and SSc patients, no functional indices differed between the two groups, with the exception of LV mass, which was significantly higher in the myocarditis group compared with SSc (82.2 (64.6−108.6) vs. 67.3 (58.0−80.0), *p* < 0.001). Regarding tissue characterization indices, T2-signal ratio was on average significantly higher in myocarditis patients compared with SSc (2.5 (0.6) vs. 2.1 (0.4), *p* < 0.001). EGE, LGE and T2-mapping did not differ significantly between the two groups. However, both native T1-mapping and ECV were on average significantly lower in the myocarditis group compared with the SSc group (1051.0 (1027.0, 1099.0) vs. 1120.0 (1065.0, 1170.0), *p* < 0.001 and 28.0 (26.0, 30.0) vs. 31.5 (30.0, 33.0), *p* < 0.001, respectively). 

### 3.3. Comparison of Cut-off Values and Lake Louise Criteria Between Groups

**Figure 1:**
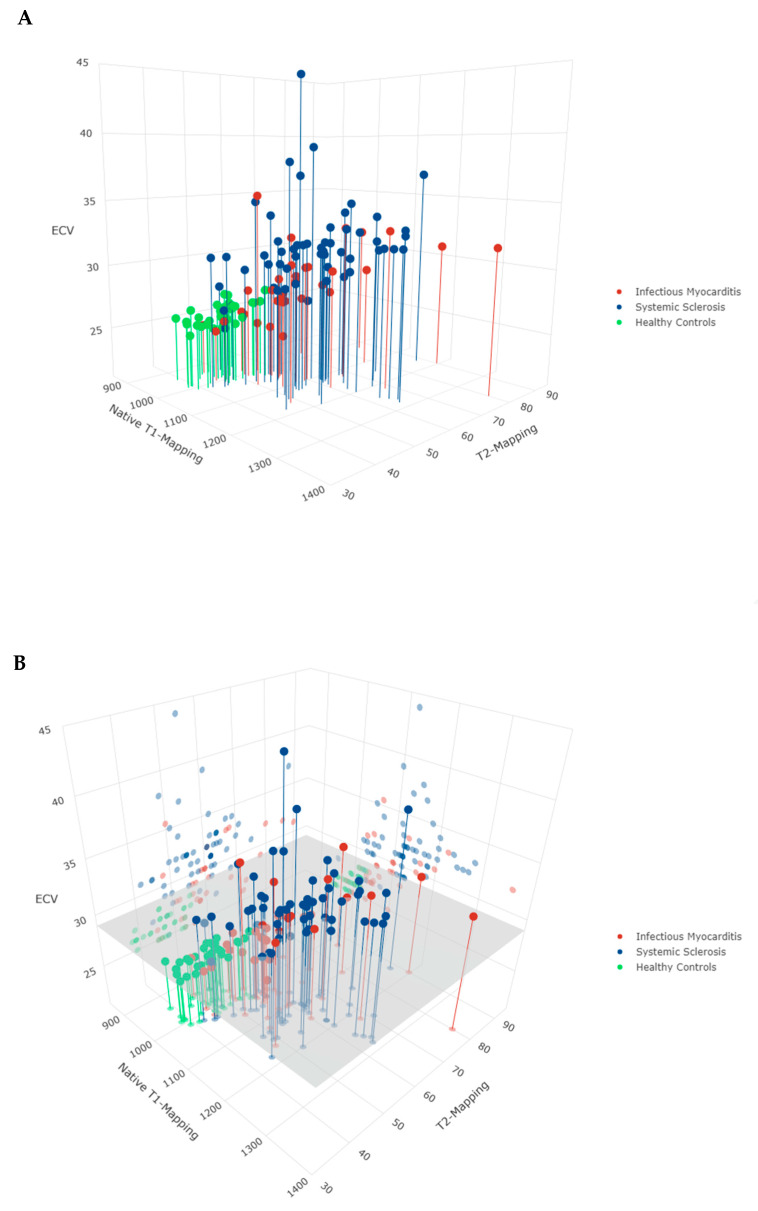
(**A**) A three-dimensional scatterplot of native T1-mapping (ms), T2-mapping (ms) and extracellular volume fraction (ECV) (%) values, colored by patient group. For each pairwise combination the corresponding point is projected on a side of the graph. (**B**) The same three-dimensional scatterplot with the addition of a horizontal plane at the pathologic cut-off value for ECV measurements used in this study (>29%). An interactive version of this plot is included as Supplementary Materials. ms milliseconds

**Figure 2:**
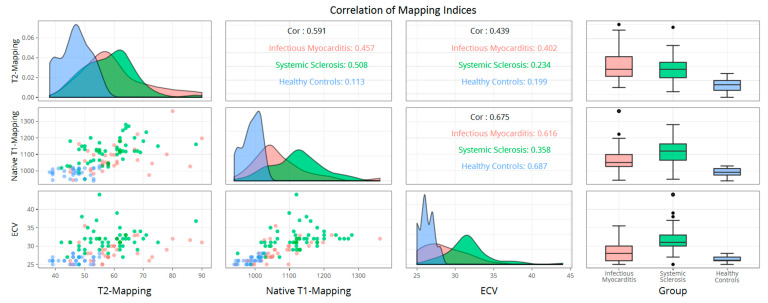
Two-dimensional scatterplots and boxplots for native T1-mapping, T2-mapping and ECV with individuals color coded according to the study group they belonged to. Spearman’s correlation coefficients are presented for each combination of the three indices collectively and for each group separately. The distribution of each variable per group is also presented as a density plot. ECV: extracellular volume fraction.

Based on locally used cut-off values, the proportion of myocarditis and SSc patients with pathologic EGE, LGE and T2-mapping did not differ significantly (Table 2). The proportion of myocarditis patients with pathologic T2-ratio was significantly higher than that of SSc patients (33 (97%) vs. 38 (64%), *p* < 0.001). Similar to when examining their average values across groups, a significantly smaller proportion of myocarditis patients had pathologic native T1-mapping and ECV values compared with SSc (17 (50%) vs. 47 (80%), *p* < 0.001 and 15 (44%) vs. 52 (90%), *p* < 0.001, respectively). Native T1-mapping, T2-mapping and ECV are plotted in two-dimensional and three-dimensional scatter plots (Figure 1 and Figure 2 respectively). At least one T1-based index was pathologic in 33 (97%) and 57 (98%) of myocarditis and SSc patients respectively (*p* = 0.699). In contrast, the proportion of myocarditis patients with at least one pathologic T2-based index was significantly higher than that of SSc patients (33 (97%) vs. 45 (76%), *p* = 0.009]. The oLLc diagnosed myocarditis in 34 (100%) myocarditis patients and 40 (69%) SSc patients, while the uLLc diagnosed 32 (94%) and 44 (76%) patients, respectively (*p* < 0.001 for both). The agreement between the oLLc and uLLc for each of the three groups is presented in Table 3. Collectively both criteria had good agreement with the most difference seen in SSc patients. Nevertheless, this did not reach statistical significance. 

### 3.4. Penalized Regression Analysis

Results of MCP penalized regression analyses for discriminating between controls and SSc/infectious myocarditis patients are presented in Table 4. The oLLc were the most important index for differentiating between controls and each group in both cases. Additionally, native T1-mapping and LV mass were also selected as features unique to the differentiation of controls form SSc patients. However, only native T1-mapping seems to have real significance seeing as LV mass had a very high mFDR with comparatively small effect size. 

### 3.5. Sensitivity Analysis

Because healthy controls more closely resembled the age and sex distribution of myocarditis patients rather than SSc patients, a cohort of 29 additional sex- and age-matched healthy controls for the SSc group (mean age, 48 ± 15 years; 24 (82.8%) females), without known cardiovascular comorbidities/risk factors, was separately used for sensitivity analyses. However, this did not lead to changes in the aforementioned findings, neither in conventional analyses nor in penalized regression analysis. Additionally, no controls were diagnosed with myocarditis according to both the oLLc and uLLc.

## 4. Discussion

In this study we present for the first time a head-to-head comparison of healthy controls, symptomatic patients with suspected infectious myocarditis and symptomatic SSc patients with suspected cardiac inflammation using a comprehensive CMR analysis. We demonstrate that infectious myocarditis patients more often had evidence of an acute inflammatory process on CMR examination (at least one pathologic T2-based index) than symptomatic SSc patients, even though almost all patients from either group had at least one pathologic T1-based index. Additionally, we demonstrate that the oLLC and uLLC had high agreement in all groups, with only minor variations in SSc patients, which nevertheless were not statistically significant. Penalized regression analyses selected the oLLc both for differentiating between controls and SSc patients as well as controls and myocarditis. 

An important finding of our study is that T2-based indices were in general significantly higher in myocarditis patients compared with SSc patients. Additionally, we demonstrate that an acute inflammatory process could not be identified in ~25% of symptomatic SSc patients, while almost all myocarditis patients had at least one pathologic T2-based index. This is in line with the respective pathophysiology of each disease. Infectious myocarditis starts by definition as an acute inflammatory process characterized by direct cardiomyocyte injury, with concomitant interstitial myocardial oedema and/or necrosis [25]. In contrast, as described previously, the cardiac effects of SSc may either result from acute myocardial inflammation similar to infectious myocarditis, or from chronic low-grade inflammation superimposed on a vasculopathy background [4]. This divergence in the chronicity of the two types of SSc-related cardiac manifestations may explain the discrepancy in T2-based indices between the two groups. These findings imply that the treatment approach to a subset of symptomatic SSc patients with suspected cardiac involvement might require reevaluation. Although not much literature is available with regard to the treatment of symptomatic patients with nonacute cardiac involvement, the vasculopathy component in these patients might best be tackled with calcium channel blockers which have been shown to improve myocardial perfusion [26]. 

Next to the clinical implications of this investigation, the relative importance of T1- and T2-based indices in symptomatic patients with infectious myocarditis and SSc merits additional discussion. Native T1 values may be affected by numerous factors including expansion of the extracellular space due to interstitial oedema or fibrosis [27]. Specifically for myocarditis, during the acute phase in which edema is most prevalent, native T1-mapping offers both excellent diagnostic sensitivity and specificity when the optimal cutoff is used [28,29,30]. However, as the early inflammation attenuates and subsequent fibrosis begins to set in, native T1-mapping prolongation gradually loses its specificity for myocardial inflammation [31,32]. In fact, in patients with symptoms lasting longer than two weeks, T2-mapping was shown to be the only CMR index that had acceptable agreement with endomyocardial biopsy for the diagnosis of myocarditis [31]. This is particularly important in SSc patients who experience more longstanding disease progression by comparison. Additionally, T2-mapping was shown to be the optimal CMR index for assessing disease activity in myocarditis [33]. In both studies, T2-mapping was compared with and was thus reportedly superior to native T1-mapping, ECV, EGE/LGE, and T2 ratio for predicting their respective endpoints. In our study population, the vast majority of both myocarditis and SSc patients had increases in at least one T1-based index. We observe that myocarditis patients had a greater prevalence of pathologic LGE values than SSc patients, with the reverse being true for native T1-mapping and ECV. Interestingly, infectious myocarditis patients did not a have a significantly higher prevalence of abnormal T2-mapping values, but had significantly higher and more often pathologic T2 ratios compared with SSc patients. This finding is in disagreement with the aforementioned studies, which suggest that T2 ratio is inferior to T2-mapping. We attribute this difference in findings to the higher slice thickness and the type of coil used for STIR-T2 image acquisition, which allow for more accurate results [34]. The fact that no controls were found to have pathologic T2 ratios lends credence to the validity of this method. 

Similar considerations as for native T1-mapping apply to ECV, which is commonly used as a surrogate marker for diffuse fibrosis [35]. Namely, myocardial inflammation has also in this case been shown to confound the correlation between ECV and fibrosis [36]. As such, the same limitations apply to ECV. Collectively, our findings are in agreement with our initial hypothesis that T1-based indices should not by themselves be considered as indicators of myocardial inflammation in SSc. Since values of T1-based indices may be confounded by expansion of the extracellular space associated with the deposition of fibrous tissue, it is not certain whether oedema or fibrosis is causing these pathologic values to appear. As such, they are not sensitive enough to distinguish between the distinct processes of acute myocardial inflammation and microvasculopathy in SSc patients, when used by themselves. In addition, various factors including differences in sequences, lack of standardization/normal values, and partial dependence on heart rate can influence the values of native T1 [37]; ECV, however, is less bound by these limitations. 

Lastly, in our population the oLLc and uLLc appeared to have very good agreement, both in healthy controls and in myocarditis/SSc patients. Both criteria were highly specific and correctly classified all healthy controls as not having myocarditis. The greatest discordance was observed in SSc patients, but did not ultimately reach statistical significance. Individual constituents of the uLLc were not demonstrated to have highly divergent diagnostic value from the oLLc in a recent meta-analysis [38]. To our knowledge, since the publication of the uLLc, only a single study has reported comparisons between them and the oLLc, mainly citing higher sensitivity and specificity for the uLLc [39]. However, since the criteria were not compared with a diagnostic gold standard, the use of the terms sensitivity and specificity are not appropriate [40]. Instead, (positive/negative) percent agreement and indices of between-method agreement as presented in our investigation constitute the appropriate inferential statistical methods in this case [40,41]. This difference in findings could again be attributed to the manner in which T2 ratios are calculated at our center. Interestingly, penalized regression analyses selected the oLLc and not the uLLc as features for differentiating between controls and SSc/myocarditis patients. Collectively, these findings suggest that not much additive value is provided by uLLc in their current form. However, these findings should be independently validated in future studies. 

### Limitations

This study did not incorporate evaluation with endomyocardial biopsy. However, this is an invasive procedure prone to sampling error and our SSc patients did not meet the currently existing indications for this investigation. In addition, each center is advised to generate its own normal cut-off values for parametric CMR indices [37]. As a result, the findings presented in this manuscript cannot necessarily be generalized to other centers. This of course does not negate the implications of the findings; however, further research is needed to elucidate these issues.

## 5. Conclusion

In symptomatic SSc patients with suspected myocardial inflammation, ~25% of participants had no appreciable acute inflammatory processes as substantiated by T2-based indices. In contrast, almost all SSc patients and infectious myocarditis patients had at least one pathologic T1-based index. This investigation supports the notion that T1-based indices should not be used by themselves as surrogates of acute myocardial inflammation in SSc patients and that T2-based indices should play a more important role in determining the diagnosis and management of such patients. 

## Figures and Tables

**Table 1:** Definition of myocarditis/myocardial inflammation based on the original and updated Lake Louise criteria. The original criteria evaluate a single variable for each of the domains of myocardial oedema, fibrosis and hyperemia. The updated criteria do not take myocardial hyperemia into account and instead only make use of one pathological T1-based index and one pathological T2-based index for the domains of myocardial fibrosis and oedema respectively.

Criterion	Myocardial Oedema		Myocardial Fibrosis		Myocardial Hyperemia		Diagnosis of Myocarditis
Original Lake Louise Criteria	STIR-T2 myocardium to skeletal muscle ratio > 1.9 1 point	+	Presence of nonischemic LGE 1 point	+	EGE > 4% 1 point	=	Myocarditis if the sum of all points from each category is ≥ 2
Updated Lake Louise Criteria	At least one abnormal T2-based index (T2-mapping > 55 ms, STIR-T2 myocardium to skeletal muscle ratio > 1.9)	+	At least one abnormal T1-based index (native T1-mapping > 1050 ms, elevated ECV>29%, presence of nonischemic LGE)		N/A		Myocarditis if at least one index in each category is pathologic

STIR: short-tau inversion recovery; EGE: early gadolinium enhancement; LGE: late gadolinium enhancement; ECV: extracellular volume fraction.

**Table 2:** Characteristics and descriptive statistics for the whole cohort in subgroups (infectious myocarditis, SSc, controls). Statistical significance for univariate statistical testing is presented.

Variable	Infectious Myocarditis	Scleroderma	Healthy Controls	*p*-Value
Number of participants	34	59	31	N/A
Demographics				
Female Sex **	15 (44%)	55 (93%)	12 (39%)	<0.001 *
Age **	38.0 (17.0)	53.6 (12.9)	39.4 (8.5)	<0.001 *
Years since SSc diagnosis	N/A	4.0 (2.0, 10.0)	N/A	N/A
Functional Indices				
LVEDV (mL)	131.4 (111.0, 165.4)	126.0 (107.0, 147.0)	119.0 (105.0, 123.0)	0.019 *
LVESV (mL)	49.7 (39.0, 67.0)	45.0 (34.0, 68.0)	36.0 (33.0, 44.0)	0.003 *
LVEF (%)	61.7 (59.7, 66.4)	63.0 (58.0, 69.0)	68.0 (65.0, 69.0)	<0.001 *
LVM ** (g)	82.2 (64.6, 108.6)	67.3 (58.0, 80.0)	81.0 (78.0, 89.0)	<0.001 *
RVEDV (mL)	131.5 (109.7, 174.0)	124.0 (99.0, 154.0)	91.0 (88.0, 98.0)	<0.001 *
RVESV (mL)	47.5 (38.0, 69.8)	45.0 (33.0, 68.0)	34.0 (32.0, 37.0)	<0.001 *
RVEF (%)	61.8 (60.0, 65.0)	61.0 (53.0, 66.0)	67.0 (66.0, 69.0)	<0.001 *
D-shaped interventricular septum	N/A	9 (15%)	N/A	N/A
Tissue Characterization Indices				
T2 ratio **	2.5 (0.6)	2.1 (0.4)	1.5 (0.2)	<0.001 *
EGE (%)	2.2 (1.8, 4.6)	3.4 (2.0, 5.1)	2.7 (1.9, 3.0)	0.050 *
LGE (%)	5.0 (5.0, 7.0)	5.0 (3.0, 6.0)	0.0 (0.0, 0.0)	<0.001 *
T2-mapping (ms)	58.0 (53.0, 67.0)	58.0 (52.0, 63.0)	47.0 (43.0, 50.0)	<0.001 *
Native T1-mapping (ms) **	1051.0 (1027.0, 1099.0)	1120.0 (1065.0, 1170.0)	992.0 (975.0, 1015.0)	<0.001 *
Post-contrast T1-mapping (ms) **	407.0 (358.0, 450.0)	348.5 (320.0, 417.0)	457.0 (434.0, 470.0)	<0.001 *
ECV (%) **	28.0 (26.0, 30.0)	31.5 (30.0, 33.0)	26.0 (26.0, 27.0)	<0.001 *
Local Cut-offs for Tissue Characterization Indices				
LGE > 0%	33 (97%)	50 (86%)	0 (0%)	<0.001 *
EGE > 4%	13 (38%)	26 (45%)	0 (0%)	<0.001 *
T2 Ratio >1.9 **	33 (97%)	38 (64%)	0 (0%)	<0.001 *
T2 Mapping > 55	24 (71%)	35 (59%)	0 (0%)	<0.001 *
Nat. T1 Mapping > 1050 ms **	17 (50%)	47 (80%)	0 (0%)	<0.001 *
Post-contrast. T1 Mapping < 350 ms **	8 (24%)	29 (50%)	0 (0%)	<0.001 *
ECV > 29% **	15 (44%)	52 (90%)	0 (0%)	<0.001 *
Original Lake Louise Criteria [17]				
Number of pathologic indices **:				
0	0 (0%)	2 (3%)	31 (100%)	
1	0 (0%)	16 (28%)	0 (0%)	<0.001 *
2	23 (68%)	22 (38%)	0 (0%)	
3	11 (32%)	18 (31%)	0 (0%)	
Diagnosis of Myocarditis/Myocardial Inflammation **	34 (100%)	40 (69%)	0 (0%)	<0.001 *
Updated Lake Louise Criteria				
At least 1 abnormal T1-based index	33 (97%)	57 (98%)	0 (0%)	<0.001 *
At least 1 abnormal T2-based index **	33 (97%)	45 (76%)	0 (0%)	<0.001 *
Diagnosis of Myocarditis/Myocardial Inflammation **	32 (94%)	44 (76%)	0 (0%)	<0.001 *

CMR: cardiovascular magnetic resonance; SSc: systemic sclerosis; LVEDV: left ventricular end diastolic volume; LVESV: left ventricular end systolic volume; LVEF: left ventricular ejection fraction; LVM: left ventricular mass; RVEDV: right ventricular end diastolic volume; RVESV: right ventricular end systolic volume; RVEF: right ventricular ejection fraction; EGE: early gadolinium enhancement; LGE: late gadolinium enhancement; ECV: extracellular volume fraction; *: *p* ≤ 0.05; **: differs significantly between infectious myocarditis and SSc in post hoc analysis.

**Table 3:** Cross-tabulations and inter-method agreement and marginal homogeneity testing between the original and updated Lake Louise criteria, denoting the presence/absence of myocarditis or myocardial inflammation (no/yes). The criteria are presented in detail in Table 1. Positive and negative agreement refer to the respective category of the oLLc. In cases where there was perfect agreement, confidence intervals for percentage agreement and some agreement statistics cannot be calculated.

	Patient Group		uLLc		Raw Agreement	Positive Agreement	Negative Agreement	McNemar’s Test	Cohen’s Kappa	Cohen’s Kappa *p*-Value
			No	Yes						
oLLc	Infectious Myocarditis	No	0	0	94.1% (80.3–99.3%)	94.1% (80.3–99.3%)	100%	0.157	N/A	N/A
		Yes	2	32						
	SSc	No	11	7	82.8% (70.6–91.4%)	92.5% (79.6–98.4%)	61.1% (35.7–82.7%)	0.164	0.57 (0.35-0.77)	<0.0001 *
		Yes	3	37						
	HC	No	31	0	100%	100%	100%	N/A	N/A	N/A
		Yes	0	0						

oLLc: original Lake Louise Criteria; uLLc: updated Lake Louise Criteria; SSc: systemic sclerosis; HC: healthy controls.

**Table 4:** Results of MCP logistic regression analysis for discriminating between controls and SSc/infectious myocarditis patients.

Comparison	Variable	Estimate	Odds Ratio	mFDR	R^2^	Signal-to-Noise Ratio	Prediction Error
Controls vs. SSc	oLLc	10.23	27,700	<0.0001 *	0.63	1.69	0.056
	Native T1-Mapping	0.53	200	<0.0001 *			
	LV Mass	−0.0087	0.991	0.999			
Controls vs. Myocarditis	oLLc	4.64	103.5	<0.0001 *	0.70	2.31	<0.001

MCP minmax concave penalty; SSc systemic sclerosis; oLLc original Lake Louise criteria; mFDR marginal false discovery rate. *: mFDR ≤ 0.05.

## Supplementary Materials

The following are available online at https://www.mdpi.com/2075-4418/10/5/335/s1, Video S1: tAn interactive 3D-graphic of the three-dimensional scatterplot presented in Figure 1. ECV extracellular volume fraction.

## References

[1] Varga J., Abraham D. (2007). Systemic sclerosis: A prototypic multisystem fibrotic disorder. J. Clin. Investig..

[2] Rubio-Rivas M., Royo C., Simeón C.P., Corbella X., Fonollosa V. (2014). Mortality and survival in systemic sclerosis: Systematic review and meta-analysis. Semin. Arthritis Rheum..

[3] Steen V.D., Medsger T.A. (2007). Changes in causes of death in systemic sclerosis, 1972–2002. Ann. Rheum. Dis..

[4] Pieroni M., De Santis M., Zizzo G., Bosello S., Smaldone C., Campioni M., De Luca G., Laria A., Meduri A., Bellocci F. (2014). Recognizing and treating myocarditis in recent-onset systemic sclerosis heart disease: Potential utility of immunosuppressive therapy in cardiac damage progression. Semin. Arthritis Rheum..

[5] Mueller K.A.L., Mueller I.I., Eppler D., Zuern C.S., Seizer P., Kramer U., Koetter I., Roecken M., Kandolf R., Gawaz M. (2015). Clinical and histopathological features of patients with systemic sclerosis undergoing endomyocardial biopsy. PLoS ONE.

[6] Mavrogeni S.I., Bratis K., Karabela G., Spiliotis G., van Wijk K., Hautemann D., Reiber J.H.C., Koutsogeorgopoulou L., Markousis-Mavrogenis G., Kolovou G. (2015). Cardiovascular Magnetic Resonance Imaging clarifies cardiac pathophysiology in early, asymptomatic diffuse systemic sclerosis. Inflamm. Allergy Drug Targets.

[7] Mavrogeni S., Karabela G., Koutsogeorgopoulou L., Stavropoulos E., Katsifis G., Plastiras S.C., Kitas G.D., Panopoulos S., Pentazos G., Tzatzaki E. (2016). Pseudo-infarction pattern in diffuse systemic sclerosis. Evaluation using cardiovascular magnetic resonance. Int. J. Cardiol..

[8] Mavrogeni S., Gargani L., Pepe A., Monti L., Markousis-Mavrogenis G., De Santis M., De Marchi D., Koutsogeorgopoulou L., Karabela G., Stavropoulos E. (2019). Cardiac magnetic resonance predicts ventricular arrhythmias in scleroderma: The Scleroderma Arrhythmia Clinical Utility Study (SAnCtUS). Rheumatology.

[9] Hachulla A.L., Launay D., Gaxotte V., De Groote P., Lamblin N., Devos P., Hatron P.Y., Beregi J.P., Hachulla E. (2009). Cardiac magnetic resonance imaging in systemic sclerosis: A cross-sectional observational study of 52 patients. Ann. Rheum. Dis..

[10] Tzelepis G.E., Kelekis N.L., Plastiras S.C., Mitseas P., Economopoulos N., Kampolis C., Gialafos E.J., Moyssakis I., Moutsopoulos H.M. (2007). Pattern and distribution of myocardial fibrosis in systemic sclerosis: A delayed enhanced magnetic resonance imaging study. Arthritis Rheum..

[11] Ferreira V.M., Piechnik S.K., Dall’Armellina E., Karamitsos T.D., Francis J.M., Ntusi N., Holloway C., Choudhury R.P., Kardos A., Robson M.D. (2013). T1 Mapping for the diagnosis of acute myocarditis using CMR: Comparison to T2-Weighted and late gadolinium enhanced imaging. JACC Cardiovasc. Imaging.

[12] Ferreira V.M., Piechnik S.K., Dallarmellina E., Karamitsos T.D., Francis J.M., Choudhury R.P., Friedrich M.G., Robson M.D., Neubauer S. (2012). Non-contrast T1-mapping detects acute myocardial edema with high diagnostic accuracy: A comparison to T2-weighted cardiovascular magnetic resonance. J. Cardiovasc. Magn. Reson..

[13] Montant P., Sigovan M., Revel D., Douek P. (2015). MR imaging assessment of myocardial edema with T2 mapping. Diagn. Interv. Imaging.

[14] Piechnik S.K., Ferreira V.M., Dall’Armellina E., Cochlin L.E., Greiser A., Neubauer S., Robson M.D. (2010). Shortened Modified Look-Locker Inversion recovery (ShMOLLI) for clinical myocardial T1-mapping at 1.5 and 3 T within a 9 heartbeat breathhold. J. Cardiovasc. Magn. Reson..

[15] Rodríguez-Reyna T.S., Morelos-Guzman M., Hernández-Reyes P., Montero-Duarte K., Martínez-Reyes C., Reyes-Utrera C., La Madrid J.V., Morales-Blanhir J., Núñez-Álvarez C., Cabiedes-Contreras J. (2014). Assessment of myocardial fibrosis and microvascular damage in systemic sclerosis by magnetic resonance imaging and coronary angiotomography. Rheumatology (United Kingdom).

[16] Radunski U.K., Lund G.K., Stehning C., Schnackenburg B., Bohnen S., Adam G., Blankenberg S., Muellerleile K. (2014). CMR in patients with severe myocarditis: Diagnostic value of quantitative tissue markers including extracellular volume imaging. JACC Cardiovasc. Imaging.

[17] Friedrich M.G., Sechtem U., Schulz-Menger J., Holmvang G., Alakija P., Cooper L.T., White J.A., Abdel-Aty H., Gutberlet M., Prasad S. (2009). Cardiovascular Magnetic Resonance in Myocarditis: A JACC White Paper. J. Am. Coll. Cardiol..

[18] Mavrogeni S., Apostolou D., Argyriou P., Velitsista S., Papa L., Efentakis S., Vernardos E., Kanoupaki M., Kanoupakis G., Manginas A. (2017). T1 and T2 Mapping in Cardiology: “mapping the Obscure Object of Desire”. Cardiology.

[19] Ferreira V.M., Schulz-Menger J., Holmvang G., Kramer C.M., Carbone I., Sechtem U., Kindermann I., Gutberlet M., Cooper L.T., Liu P. (2018). Cardiovascular Magnetic Resonance in Nonischemic Myocardial Inflammation: Expert Recommendations. J. Am. Coll. Cardiol..

[20] Breheny P., Huang J. (2011). Coordinate descent algorithms for nonconvex penalized regression, with applications to biological feature selection. Ann. Appl. Stat..

[21] Miller R.E., Breheny P. (2019). Marginal false discovery rate control for likelihood-based penalized regression models. Biometrical J..

[22] Breheny P.J. (2019). Marginal false discovery rates for penalized regression models. Biostatistics.

[23] Liu H., Du G., Zhang L., Lewis M.M., Wang X., Yao T., Li R., Huang X. (2016). Folded concave penalized learning in identifying multimodal MRI marker for Parkinson’s disease. J. Neurosci. Methods.

[24] Mehta P., Bukov M., Wang C.H., Day A.G.R., Richardson C., Fisher C.K., Schwab D.J. (2019). A high-bias, low-variance introduction to Machine Learning for physicists. Phys. Rep..

[25] Friedrich M.G., Marcotte F. (2013). Cardiac magnetic resonance assessment of myocarditis. Circ. Cardiovasc. Imaging.

[26] Bournia V.K., Tountas C., Protogerou A.D., Panopoulos S., Mavrogeni S., Sfikakis P.P. (2018). Update on assessment and management of primary cardiac involvement in systemic sclerosis. J. Scleroderma Relat. Disord..

[27] Taylor A.J., Salerno M., Dharmakumar R., Jerosch-Herold M. (2016). T1 Mapping Basic Techniques and Clinical Applications. JACC Cardiovasc. Imaging.

[28] Luetkens J.A., Homsi R., Sprinkart A.M., Doerner J., Dabir D., Kuetting D.L., Block W., Andrié R., Stehning C., Fimmers R. (2016). Incremental value of quantitative CMR including parametric mapping for the diagnosis of acute myocarditis. Eur. Heart J. Cardiovasc. Imaging.

[29] Hinojar R., Foote L., Ucar E.A., Jackson T., Jabbour A., Yu C.Y., McCrohon J., Higgins D.M., Carr-White G., Mayr M. (2015). Native T1 in discrimination of acute and convalescent stages in patients with clinical diagnosis of myocarditis: A proposed diagnostic algorithm using CMR. JACC Cardiovasc. Imaging.

[30] Luetkens J.A., Doerner J., Thomas D.K., Dabir D., Gieseke J., Sprinkart A.M., Fimmers R., Stehning C., Homsi R., Schwab J.O. (2014). Acute myocarditis: Multiparametric cardiac MR imaging. Radiology.

[31] Lurz P., Luecke C., Eitel I., Föhrenbach F., Frank C., Grothoff M., De Waha S., Rommel K.P., Lurz J.A., Klingel K. (2016). Comprehensive Cardiac Magnetic Resonance Imaging in Patients with Suspected Myocarditis the MyoRacer-Trial. J. Am. Coll. Cardiol..

[32] Von Knobelsdorff-Brenkenhoff F., Schüler J., Dogangüzel S., Dieringer M.A., Rudolph A., Greiser A., Kellman P., Schulz-Menger J. (2017). Detection and Monitoring of Acute Myocarditis Applying Quantitative Cardiovascular Magnetic Resonance. Circ. Cardiovasc. Imaging.

[33] Bohnen S., Radunski U.K., Lund G.K., Kandolf R., Stehning C., Schnackenburg B., Adam G., Blankenberg S., Muellerleile K. (2015). Performance of T1 and T2 Mapping Cardiovascular Magnetic Resonance to Detect Active Myocarditis in Patients with Recent-Onset Heart Failure. Circ. Cardiovasc. Imaging.

[34] Abdel-Aty H., Boyé P., Zagrosek A., Wassmuth R., Kumar A., Messroghli D., Bock P., Dietz R., Friedrich M.G., Schulz-Menger J. (2005). Diagnostic performance of cardiovascular magnetic resonance in patients with suspected acute myocarditis: Comparison of different approaches. J. Am. Coll. Cardiol..

[35] Haaf P., Garg P., Messroghli D.R., Broadbent D.A., Greenwood J.P., Plein S. (2016). Cardiac T1 Mapping and Extracellular Volume (ECV) in clinical practice: A comprehensive review. J. Cardiovasc. Magn. Reson..

[36] Lurz J.A., Luecke C., Lang D., Besler C., Rommel K.P., Klingel K., Kandolf R., Adams V., Schöne K., Hindricks G. (2018). CMR–Derived Extracellular Volume Fraction as a Marker for Myocardial Fibrosis: The Importance of Coexisting Myocardial Inflammation. JACC Cardiovasc. Imaging.

[37] Messroghli D.R., Moon J.C., Ferreira V.M., Grosse-Wortmann L., He T., Kellman P., Mascherbauer J., Nezafat R., Salerno M., Schelbert E.B. (2017). Clinical recommendations for cardiovascular magnetic resonance mapping of T1, T2, T2 and extracellular volume: A consensus statement by the Society for Cardiovascular Magnetic Resonance (SCMR) endorsed by the European Association for Cardiovascular Imagin. J. Cardiovasc. Magn. Reson..

[38] Pan J.A., Lee Y.J., Salerno M. (2018). Diagnostic Performance of Extracellular Volume, Native T1, and T2 Mapping Versus Lake Louise Criteria by Cardiac Magnetic Resonance for Detection of Acute Myocarditis: A Meta-Analysis. Circ. Cardiovasc. Imaging.

[39] Luetkens J.A., Faron A., Isaak A., Dabir D., Kuetting D., Feisst A., Schmeel F.C., Sprinkart A.M., Thomas D. (2019). Comparison of Original and 2018 Lake Louise Criteria for Diagnosis of Acute Myocarditis: Results of a Validation Cohort. Radiol. Cardiothorac. Imaging.

[40] United States Food and Drug Administration (2007). Statistical Guidance on Reporting Results from Studies Evaluating Diagnostic Tests—Guidance for Industry and FDA Staff. FDA.

[41] Watson P.F., Petrie A. (2010). Method agreement analysis: A review of correct methodology. Theriogenology.

